# Prostatic metastases and polycythemia vera on bone magnetic resonance imaging: A case report

**DOI:** 10.3892/ol.2014.2809

**Published:** 2014-12-17

**Authors:** YUYING LI, WEI AN, WEI WEI, MIN LIU, GUANJUN WANG, XU WANG

**Affiliations:** 1 Cancer Center, The First Hospital, Jilin University, Changchun, Jilin 130021, P.R. China; 2 Department of Urology, The First Hospital, Jilin University, Changchun, Jilin 130021, P.R. China

**Keywords:** prostate cancer, polycythemia vera, bone metastases

## Abstract

Localized prostate cancer and polycythemia vera (PV) are rare, and can be difficult to distinguish from bone marrow metastatic prostate cancer. The present study describes a case of prostate cancer occurring with PV. Magnetic resonance imaging revealed diffusely inhomogeneous bone marrow of the pelvis and a localized prostatic mass. The bone marrow biopsy indicated erythrocytosis and leukocytosis. The patient was treated with aspirin and twice-weekly phlebotomy procedures for two weeks, followed by a radical prostatectomy. Following the surgery, the patient was continually treated with interferon-α2b and aspirin, and demonstrated no abnormalities within the one-year follow-up period. The findings of the present study may aid in the future diagnosis and treatment of patients with prostate cancer and PV.

## Introduction

Prostate cancer is the most common form of cancer and the second leading cause of cancer-related mortality among males in the United States (1). In 2010, there was an incidence of ~217,000 new prostate cancer cases and 32,000 mortalities due to prostate cancer in the United States (1). Advanced-stage prostate cancer is characterized by tropism to bone, with skeletal involvement present in ~90% of patients with metastatic disease (2,3). Metastatic disease to bone is commonly associated with skeletal-related events, which are associated with decreased quality of life, increased pain and worsened survival (4). A bone scan is the standard method for detecting bone metastases from prostate cancer. In certain cases, additional tests, including computed tomography scan, magnetic resonance imaging (MRI) scan or bone biopsy, may be needed to diagnose bone metastases. Local external beam radiotherapy, systemic radioisotope therapy, endocrine therapy, chemotherapy, and bisphosphonates and denosumab are the mainstays of treatment, as well as painkillers and other usual classical interventions (5).

Polycythemia vera (PV), a type of myeloproliferative neoplasm, is a clonal disorder characterized by unwarranted production of red blood cells, and associated with JAK2 mutations (V617F or exon 12) in almost all cases (6). It is more common in the elderly and may be symptomatic or asymptomatic. Typical signs and symptoms include itching (pruritus) and severe burning pain in the hands or feet that is frequently accompanied by a red/blue coloration of the skin. Other disease features include leukocytosis, splenomegaly, thrombohemorrhagic complications, vasomotor disturbances and a small risk of disease progression into acute myeloid leukemia (AML) or myelofibrosis (MF) (7). Diagnosis of PV is currently according to World Health Organization criteria and based on a composite assessment of clinical and laboratory features, including two major criteria (increased hemoglobin levels and presence of JAK2 mutation) and three minor criteria (trilineage myeloproliferation in bone marrow biopsy, increased serum erythropoietin levels and endogenous erythroid colony formation *in vitro*) (8). The aims of therapy are to prevent the occurrence and recurrence of thrombosis; to delay (if feasible) evolution into MF or AML; and to control disease-associated symptoms (9). The treatment strategy in PV patients comprises a combination of modification of cardiovascular risk factors, antiplatelet therapy, phlebotomy and cytoreduction (9). Both of the diseases can involve bone or bone marrow, but each disease has unique changes in bone structure. As treatment options are fundamentally different between metastatic prostate cancer and PV and it is therefore important to distinguish the differences using different techniques, such as CT, MRI and bone scans. We report a case of prostate cancer and PV and our approach to differential diagnosis for these diseases using radiological imaging techniques. The patient provided written informed consent.

## Case report

A 60-year-old male was admitted to The First Hospital, Jilin University (Changchun, China) in October 2012 complaining of redness on the hands and face that had been apparent for three months. The patient exhibited no feelings of discomfort, and reported that the skin problems appeared to be insidious. A review of the medical history revealed that the patient had experienced a lacunar infarct eight years previously, and had suffered with hypertension for >30 years, for which antihypertensive drugs had been taken for ~20 years. The patient did not smoke or regularly consume alcohol. Immediate family members exhibited no history of any neurological or hematological diseases.

A physical examination revealed that the patient was afebrile, with an Eastern Cooperative Oncology Group score of 0 (10), a normal heart rate and a blood pressure of 145/90 mmHg. The face, neck and hands appeared red, but no pain was reported. There was no evidence of edema or blistering of the skin. Lymphadenopathy, hepatosplenomegaly or an abnormal physical mass in the abdomen were not apparent. Digital rectal examination did not reveal any masses or enlargement of the prostate.

Hematological analysis revealed slightly elevated readings for hemoglobin (Hb; 20.4 g/dl; normal range, 12.0–16.0 g/dl) and the hematocrit (Hct; 0.610 l/l; normal range, 0.400–0.500 l/l), and also evidence of leukocytosis [white blood cell (WBC) count, 12.89×10^9^/l; normal range, 4.0–10.0×10^9^/l]. The WBC count consisted of 80% neutrophils (normal range, 50–70%), but normal levels of platelets (253×10^9^/l; normal range, 100–300×10^9^/l). The concentration of alkaline phosphatase (ALP) was slightly high (115.0 U/l; normal range, 15.0–112.0 U/l), but liver function tests appeared normal. The serum total prostate-specific antigen (TPSA) level was 10.94 ng/ml (normal range, 0–4 ng/ml), and the serum free PSA (FPSA) level was 0.98 ng/ml. As a result, the percentage of FPSA in TPSA was 9%, which was less than the normal value of 19%.

**Figure 1:**
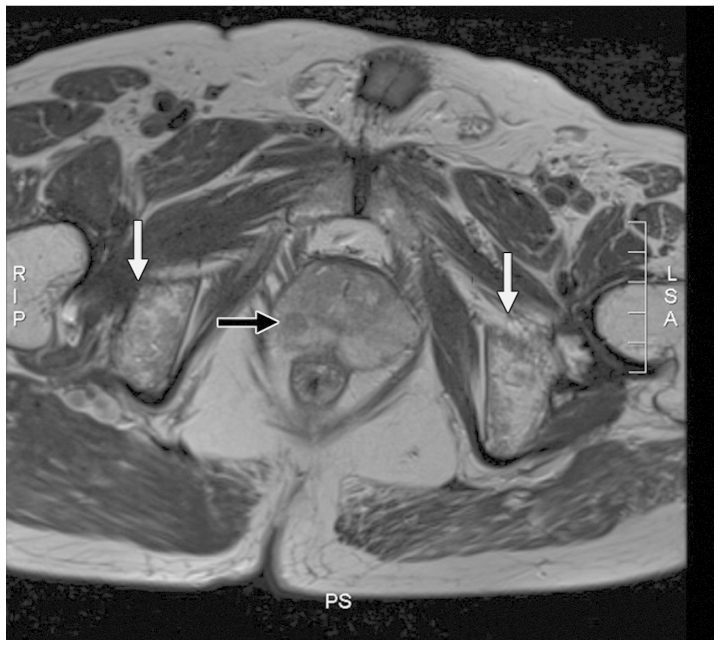
Magnetic resonance imaging of the prostate revealing a small nodule with abnormal signals located in the right prostatic peripheral zone (black arrow). The prostate capsule was continuous and complete, without lymph node enlargement. The signal intensity of the pelvic bone marrow was diffusely inhomogeneous (white arrows), and the structure of the cortical bone was intact.

MRI of the prostate (Fig. 1) revealed a small nodule measuring 5×5 mm, which exhibited abnormal intensity signals in the right prostatic peripheral zone. The capsule of the prostate gland was continuous and complete, and no evidently enlarged surrounding lymph nodes were observed. The signal intensity of the pelvic bone marrow was diffusely inhomogeneous, but the structure of the cortical region was intact. A bone scan indicated slightly elevated radioactivity within the left ankle and right knee, but without any other abnormalities. A computed tomography scan of the lung appeared normal. A transrectal ultrasound-guided biopsy of the prostate initially revealed a well-differentiated adenocarcinoma measuring <0.5×0.1 cm, with a Gleason score of 3+3 (11). The bone marrow aspiration and biopsy revealed erythrocytosis and leukocytosis consisting of 12% eosinophils (normal range, 0–8%). The genetic chromosome analysis indicated the presence of a JAK2-V617F mutation, but no other abnormalities.

A diagnosis of stage IIA prostate adenocarcinoma (TNM stage, cT1aN0M0) and PV was established (12). The patient was subjected to phlebotomy procedures to remove 400 ml of blood twice weekly for two weeks, and was treated with 100 mg aspirin daily. Prior to surgery, the blood Hb level dropped to 14.1 g/dl, and the Hct to 0.47 l/l. In November 2012, a radical prostatectomy was performed by laparoscopy. Pathological examination of the surgical specimen revealed an adenocarcinoma with negative margins, but with no evidence of seminal vesicle invasion or extra-capsular extension. The tumor volume occupied ~3% of the total prostate volume. The patient was discharged seven days after the surgery. Following surgery, the Hb level and Hct values gradually increased. The patient was therefore continually treated with 300×10^4^ units of interferon-α2b (Beijing Kawin Bio-Tech Co., Ltd., Beijing, China) twice-weekly, and 100 mg aspirin daily.

The patient was continually followed up, and the serum TPSA levels were monitored every three months. Abnormal levels of serum TPSA were not reported during the one-year follow-up period. At six months post-surgery, MRI analysis of the prostate indicated no evident abnormalities. However, there was no marked change in the atypical signal intensity of the pelvic bone marrow that was detected during the initial MRI scan. At present, the blood Hb, Hct, platelet and WBC counts remain within the normal ranges.

## Discussion

Prostate cancer is the most frequently diagnosed non-cutaneous type of cancer, and the second most common cause of cancer-related mortality affecting males in the United States. In 2010, there were an estimated 217,730 cases of newly-diagnosed prostate cancer, and at present, the disease has an estimated lifetime disease incidence in males of 20% (1). The majority of patients with advanced prostate cancer also present with bone metastases (BMs) (13–15). Patients with metastatic prostatic cancer are treated with different therapeutic strategies to those without. Therefore, it is critical to rule out the presence of metastases, such as BMs, prior to the initiation of treatment. Patients with localized prostate cancer, which is most often curable, usually benefit from radical treatment. By contrast, patients with prostate cancer and BMs are not recommended for surgery, and are usually treated with systemic chemotherapies. Several risk factors, including high levels of serum PSA (16–20), the presence of locally advanced tumors (16,20,21), high levels of serum ALP (16,22,23) and high Gleason scores (20), are believed to be associated with BMs.

In the present study, the results of the MRI scan made the diagnosis challenging. The diffusely inhomogeneous signals in the bone marrow of the pelvis, together with a small mass in the prostatic tissue, should indicate the presence of BMs. The patient exhibited histological evidence of adenocarcinoma with a Gleason score of 6, a serum PSA level of 10.94 ng/ml, a slightly high level of serum ALP and a small mass in the prostatic tissue, with a continuous and complete capsule and no evidence of lymph node enlargement. Therefore, a diagnosis of localized, early-stage prostate cancer, rather than a local BM, was proposed. Previous studies have identified that prostate cancer-related BMs usually invade the cortical bone, and rarely affect the bone marrow (24,25). Furthermore, BMs are characteristically accompanied by cytopenia, which ruled out the likelihood of BMs in the present case. Due to the importance of identifying the presence of BMs for correct diagnosis, a bone biopsy was performed in the present study, which identified no prostate cancer-related BMs. In addition, the bone scan did not reveal any evidence of BMs. Previous studies have demonstrated the presence of diffusely inhomogeneous signals in the bone marrow of patients with PV (26,27). In the present study, the results of the laboratory tests revealed an abnormal WBC count, percentage of neutrophils, and blood Hb and Hct, and the presence of a JAK-2V617F mutation. Therefore, the patient was primarily diagnosed with PV and treated with aspirin, which significantly reduced the levels of blood Hb and Hct prior to the radical prostatectomy.

According to the 2013 NCCN Guidelines for Prostate Cancer, the tumor recurrence risk is intermediate, and there are no adverse features (5). Therefore, the patient was not recommended for further aggressive post-surgical therapies. Despite no significant changes identified in the signal intensity of the pelvic bone marrow during the one-year follow-up period, abnormal levels of serum PSA were not detected, which indicated no recurrence of the prostate cancer. Upon MRI analysis, prostate cancer-related BMs are often difficult to distinguish from prostate cancer cases occurring with PV. Therefore, it is important for physicians to consider these rare diseases in clinical practice. The use of bone biopsies and systemic bone scans should allow for discrimination between the two. In addition, careful analysis of MRI, with close attention to the bone structure, together with a hematological examination, should aid in the diagnosis of PV. Empiric therapies, which in the present study included the phlebotomy procedure and aspirin treatment, are valuable as they have the potential to improve clinical symptoms, restore the hematological status and improve the condition of the body for subsequent surgical treatments. The surgical removal of prostate tumors should improve the clinical symptoms of patients with prostate cancer-related PV, however, in the present study, increased values of Hb and Hct were observed post-surgery, which indicated an incomplete response to phlebotomy and aspirin treatment. The patient was subsequently treated with interferon-α2b, which effectively controlled the symptoms of PV. Therefore, if available, interferon-α2b should be administered to patients with PV. However, the precise mechanism underlying the therapeutic action of interferon-α2b requires further investigation.

The present study described a case of prostate cancer occurring with PV. The simultaneous presence of these diseases should be recognized, and careful analysis of diagnostic imaging, such as MRI, should be performed. Furthermore, risk factors for BMs should be considered following the detection of abnormal intensity signals in the pelvis of patients with prostate cancer. To the best of our knowledge, this was the first case study to describe the systemic medical history and treatment of a patient with prostate cancer occurring with PV. The findings of the present study may aid in the future diagnosis and treatment of patients with prostate cancer and PV.
